# Effects of Carvacrol on Oxidative Stress and Fibrosis in Streptozotocin-Induced Diabetic Nephropathy: Histological, Gene Expression, and Biochemical Insights

**DOI:** 10.3390/ijms27010291

**Published:** 2025-12-27

**Authors:** Halime Tuba Canbaz, Mehmet Enes Sozen, Ilknur Cinar Ayan, Hasan Basri Savas, Furkan Adem Canbaz, Gokhan Cuce, Serpil Kalkan

**Affiliations:** 1 Department of Histology and Embryology, Hamidiye Faculty of Medicine, University of Health Sciences, 34668 Istanbul, Türkiye; 2 Department of Histology and Embryology, Faculty of Medicine, Alanya Alaaddin Keykubat University, 07425 Antalya, Türkiye; 3 Department of Medical Biology, Faculty of Medicine, Necmettin Erbakan University, 42090 Konya, Türkiye; 4 Department of Medical Biochemistry, Faculty of Medicine, Mardin Artuklu University, 47200 Mardin, Türkiye; 5 Department of Pediatric Urology, Sancaktepe Sehit Prof. Dr. İlhan Varank Training and Research Hospital, 34785 Istanbul, Türkiye; 6 Department of Histology and Embryology, Faculty of Medicine, Necmettin Erbakan University, 42090 Konya, Türkiye

**Keywords:** carvacrol, diabetes mellitus, kidney, antioxidant, histopathology

## Abstract

Diabetes mellitus (DM) leads to renal damage through oxidative stress. Carvacrol (CAR), a monoterpenoid phenol, possesses anti-inflammatory and antioxidant properties. We investigated the potential effects of CAR on histological, gene expression, and biochemical parameters in a rat model of DM. Four groups were created: group 1, control; group 2 (*n* = 9), DM; group 3 (*n* = 9), DM + dimethyl sulfoxide (DMSO); and group 4 (*n* = 9), DM + CAR. DM was created by injecting streptozotocin (STZ). CAR (20 mg/kg) was prepared through dissolution in 0.1% DMSO. CAR and 0.1% DMSO were administered daily for 4 weeks to groups 4 and 3, respectively. At the end of this study, urea, creatinine, paraoxonase-1 (PON-1), and arylesterase (ARES) were measured in serum samples. Histopathological changes and expression of Nuclear factor erythroid 2–related factor 2 (Nrf-2) in renal tissues were assessed. Immunohistochemical(ihc) staining and RT-qPCR analysis were performed to evaluate apoptosis, focusing on *Bax* and *Bcl-2*gene expression. Masson’s trichrome(MT) staining and RT-qPCR analysis of *COL1A1* and *COL3A1* mRNA levels were used to assess fibrosis. Increased urea and creatinine levels in DM were significantly decreased after CAR administration. CAR application also improved reduced levels of PON 1 and ARES, which are associated with diabetes. Both immunohistochemistry and RT-qPCR analyses revealed that CAR therapy mitigated the diabetes-induced elevation in *Bax* and reduction in *Bcl-2* expression. CAR treatment improved histopathological findings and renal Nrf-2 immunofluorescence(if) intensity. Furthermore, gene expression analysis demonstrated that *COL1A1* and *COL3A1* were upregulated in DM, while CAR administration downregulated them. In conclusion, CAR has a protective role in decreasing renal impairment linked to DM by regulating Bax and Bcl-2 levels and rectifying histological damage.

## 1. Introduction

Diabetes mellitus (DM) is a systemic disease caused by insulin deficiency. Inadequate insulin production or insulin resistance are the two leading causes of this insufficiency, which results in type 1 or type 2 DM. This condition ultimately leads to persistent hyperglycemia. An elevated concentration of glucose in the bloodstream is linked to the disruption of carbohydrate metabolism, which is regulated by enzymes [1,2]. Chronic hyperglycemia and hyperlipidemia induce oxidative stress, which facilitates the onset of diabetes sequelae, including cardiovascular illnesses, renal impairment, and retinal damage [3]. The excessive generation of reactive oxygen species (ROS) via mechanisms including mitochondrial dysfunction, NADPH oxidase activation, advanced glycation end product (AGE) synthesis, and protein kinase C signaling is crucial in the ongoing development of diabetic nephropathy (DN). ROS-induced injury affects glomerular endothelial cells, mesangial cells, and podocytes, promoting cellular apoptosis, mesangial expansion, and extracellular matrix accumulation [4]. Oxidative stress also increases the levels of profibrotic factors, especially transforming growth factor-β (TGF-β), which leads to glomerulosclerosis and tubulointerstitial fibrosis. In addition, ROS activate inflammatory pathways via redox-sensitive transcription factors, such as nuclear factor-κB, contributing to chronic inflammation and renal dysfunction [5]. Several antioxidants, including resveratrol, curcumin, α-lipoic acid (α-LA), α-tocopherol (vitamin E), vitamin C, and selenium, have been studied for their potential in treating DN via this mechanism [4]. In light of the variable or potentially modest effects reported for some antioxidant and anti-inflammatory agents, other molecules with similar properties are still under study.

CAR (2-Methyl-5-[1-methyl ethyl]-phenol), whose chemical formula is C6H3CH3 (OH) (C3H7), is the primary constituent of several essential oils derived from fragrant plants, such as musk, oregano, thyme, and thymus [6,7]. Thyme oil is an essential oil known for containing CAR among its constituents and has been utilized for therapeutic purposes for a long time. Other derivatives of thyme oil include thymol, linalool, and *p*-cymene [8]. Studies have reported the antibacterial, antifungal, antiviral, and antitumoral properties of these essential oil components [9]. Additionally, thymol and CAR have been demonstrated to aid in wound healing by enhancing cell viability and promoting tissue repair, while also preventing the onset of fibrosis and contractures [10]. In addition to its many biological benefits, including anti-inflammatory, antioxidative, anticancer, antibacterial, and anti-apoptotic activities, CAR has been demonstrated to lower blood glucose levels in mice with diabetes induced by streptozotocin (STZ) [11]. Furthermore, CAR contributes to the amelioration of diabetes-associated neurodegenerative, cognitive, vascular, cardiac, and hepatic problems by modulating blood glucose levels and mitigating oxidative stress damage [12]. However, a restricted number of studies focused on the effects of CAR in DN. The sole research in the English literature assessed oxidative stress markers and renal function tests, although it lacked histological evaluation [6]. In this context, we aimed to address this gap with the present study.

This study investigated the efficacy of CAR in mitigating oxidative stress induced by DM and the consequent renal tissue damage, employing histological, RT-qPCR, and biochemical analyses.

## 2. Results

### 2.1. Biochemical Analysis

**Figure 1:**
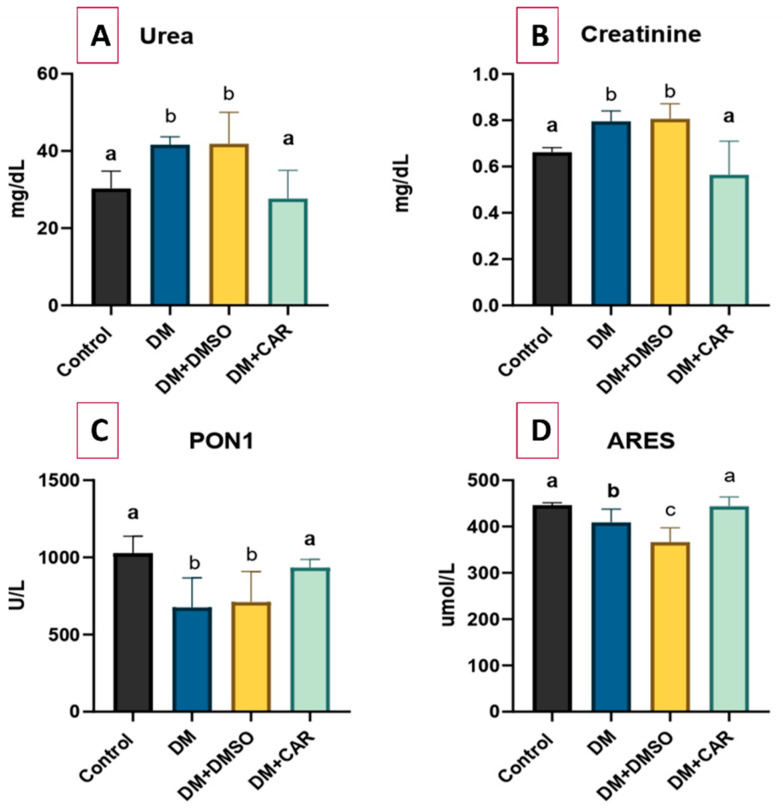
Comparison of biochemical parameters among groups; (**A**) Urea, (**B**) Creatinine, (**C**) PON1, and (**D**) ARES. There is a statistically significant difference between the groups not sharing the same letter (*p* < 0.05).

The DM group had higher levels of blood urea and creatinine than the control group, which suggests kidney damage (*p* < 0.01 for both). The DM + DMSO group also showed higher blood urea and creatinine levels compared to the control group (*p* < 0.01 for both). Conversely, the DM + CAR group demonstrated a significant decrease in both markers compared to the DM group (*p* < 0.01 for both). The levels of urea and creatinine were determined to be lower in the DM + CAR group than in the DM + DMSO group (*p* < 0.01 for both). There was no significant difference in urea and creatinine levels between the DM + CAR and control groups (*p* > 0.05 for both), indicating a potential protective effect of CAR on renal function (Figure 1A,B).

The values of the PON1 and ARES, which are antioxidant enzymes, were found to be reduced in the DM group compared to the control (*p* < 0.01 for PON1 and *p* = 0.02 for ARES). The DM + DMSO group also demonstrated lower levels of PON1 and ARES compared to the control group, with a significance level of *p* < 0.01 for both. CAR administration successfully reversed this decrease, restoring both PON1 and ARES levels to values comparable to the control group, in accordance with its antioxidant capabilities (*p* > 0.05 for both). The DM + CAR group also had higher levels of PON1 and ARES compared to the DM + DMSO group (*p* = 0.03 and *p* < 0.01, respectively) (Figure 1C,D).

### 2.2. Histopathological Analysis

**Figure 2:**
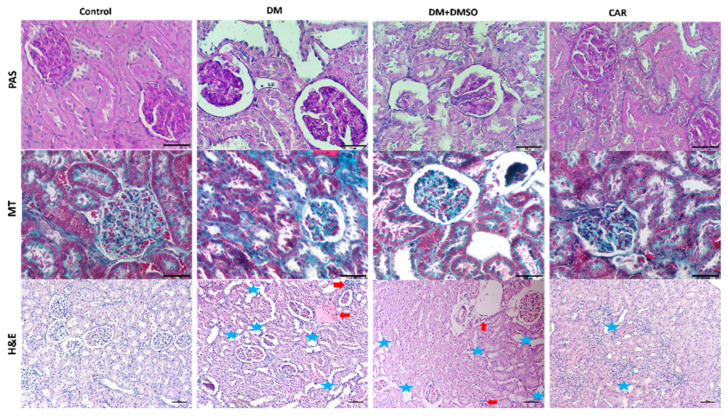
PAS, MT, and H&E staining. Blue stars show tubular dilatation, and red arrows show inflammatory cells. All bars show 50 μm (Magnification ×40 in PAS and MT, ×10 in H&E).

**Figure 3:**
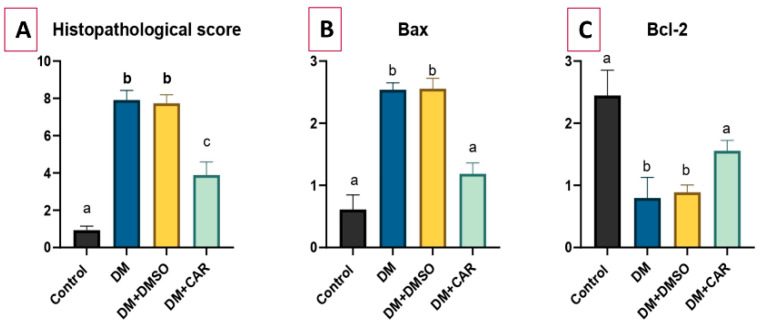
(**A**) Histopathological scores of experimental groups. Immunohistochemical scoring, including (**B**) Bax and (**C**) Bcl-2expression of groups. There is statistically significant difference between the groups not sharing the same letter (*p* < 0.05).

Hematoxylin-eosin (H&E)staining demonstrated that diabetic rats displayed significant glomerular and tubular damage, characterized by an enlarged urinary space, tubular dilatation, and infiltration of inflammatory cells. Periodic acid–Schiff (PAS) staining demonstrated glomerular basement membrane thickening and an enlarged, disordered mesangial area. All renal histological alterations induced by DM were greatly mitigated in the CAR-treated group, and fibrosis was also seen prominently in the DM and DM + DMSO groups (Figure 2, H&E staining). Histopathological scores were found to be higher in the DM and DM + DMSO groups compared to the control (*p* < 0.01 for both). The DM + CAR group had significantly lower histopathological scores compared to the DM and DM + DMSO groups (*p* < 0.01 for each comparison) (Figure 3A).

### 2.3. IHC Analysis

An increased level ofBaxexpression was determined in the DM group when compared to the control (*p* < 0.01). No significant difference was determined between the DM and DM + DMSO groups (*p* > 0.05). CAR caused decreased expression of Baxcompared to either the DM or DM + DMSO groups (*p* = 0.04 and *p* = 0.03, respectively). Moreover, no significant difference was observed between the DM + CAR and control groups (*p* > 0.05) (Figure 3B).

**Figure 4:**
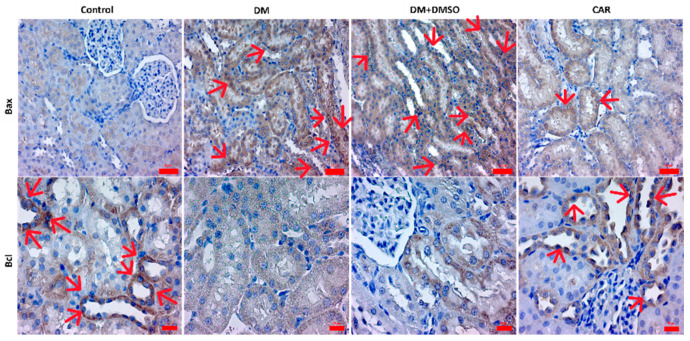
IHC staining of Bax and Bcl-2; red arrows show expressions. Bars show 50 μm in Bax staining images (magnification ×20), 20 μm in Bcl staining images (magnification ×40).

Bcl-2 expression was lower in the DM group than in the control group (*p* < 0.01). While no significant difference was observed between the DM and DM + DMSO groups (*p* > 0.05), the expression of Bcl-2was found to be increased in the DM + CAR group compared to either the DM or DM + DMSO groups (*p* = 0.02 and *p* = 0.03, respectively) (Figure 3C). There was also no significant difference between the DM + CAR and the control group (*p* > 0.05). IHC staining of Bax and Bcl-2 is presented in Figure 4.

### 2.4. IF Analysis

**Figure 5:**
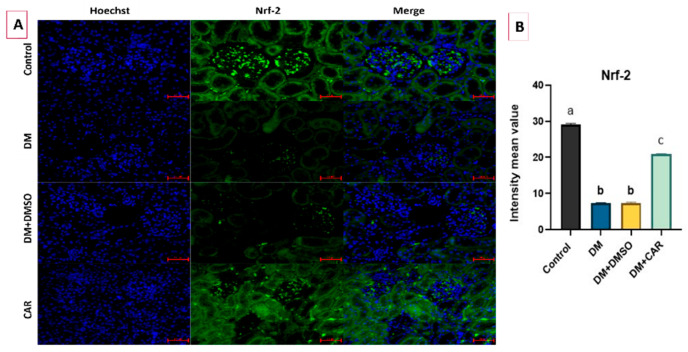
(**A**) IF staining of Nrf-2. Bars show 50 μm (magnification ×20). (**B**) Nrf-2 intensity mean values. There is statistically significant difference between the groups not sharing the same letter (*p* < 0.05).

The expression of Nrf-2in the experimental groups is depicted in Figure 5A, demonstrating the relative variability in its expression as measured via IF staining. A decreased level of the Nrf-2 expression was determined in the DM group when compared to the control (*p* < 0.01). No significant difference was determined between the DM and DM + DMSO groups (*p* > 0.05). CAR caused increased expression of Nrf-2 compared to either the DM or DM + DMSO groups (*p* < 0.01 for both) (Figure 5B).

### 2.5. RT-qPCR Analysis

The expression of the *Bax* gene was significantly increased by 4.26-fold (*p* < 0.01) in the DM-treated group compared to the control group. Conversely, a 2.06-fold increase in *Bax* gene expression was noted in the DM + CAR group compared to the control; yet, this difference was not significant (*p* > 0.05). The gene expression levels in the DM + DMSO group were comparable to those in the DM group for all analyzed genes. Moreover, in comparison to the DM group, *Bax* gene expression in the DM + CAR group was significantly reduced by 2.28-fold (*p* = 0.02).

Compared to the control group, *Bcl-2* mRNA expression decreased significantly by 6.8-fold in the DM group (*p* < 0.01) and by 1.73-fold in the DM + CAR group (*p* = 0.02). In comparison to the DM group, the DM + CAR group demonstrated a 4.11-fold elevation in *Bcl-2* expression (*p* = 0.02).

A 4.78-fold increase in *COL1A1* mRNA expression was observed in the DM-treated group compared to the control (*p* < 0.01), whereas the 1.74-fold increase detected in the DM + CAR group was not statistically significant (*p* > 0.05). In comparison to the DM group, *COL1A1* expression in the DM + CAR group was significantly diminished by 2.75-fold (*p* < 0.01).

**Figure 6:**
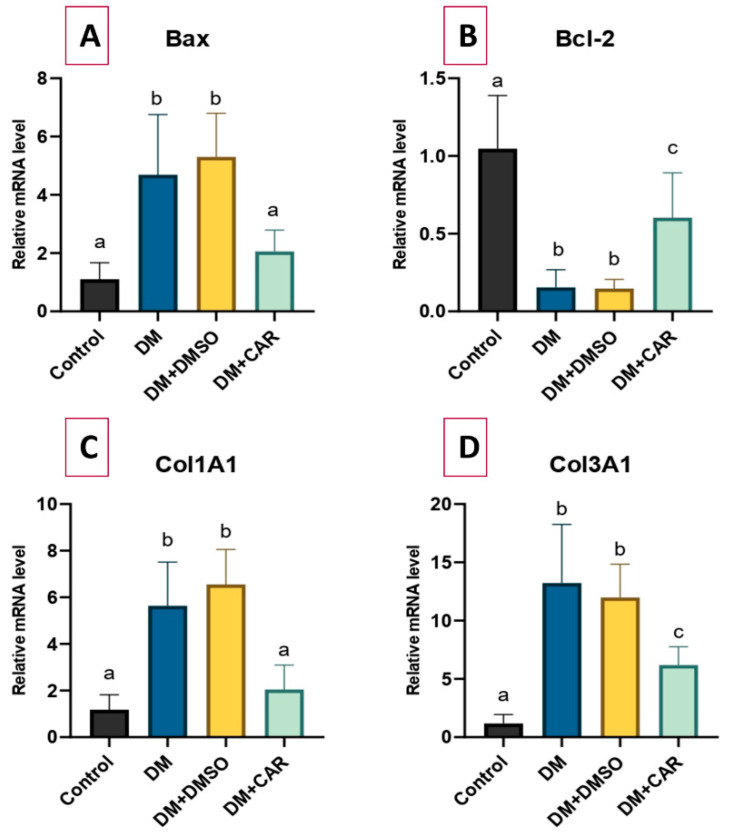
RT-qPCR analysis of the mRNA expression level of (**A**) *Bax*, (**B**) *Bcl-2*, (**C**) *COL1A1*, and (**D**) *COL3A1* among groups. There is statistically significant difference between the groups not sharing the same letter (*p* < 0.05).

Analysis of *COL3A1* mRNA expression demonstrated an 11.17-fold increase in the DM group relative to the control (*p* < 0.01), whereas a 5.23-fold rise was noted in the DM + CAR group (*p* = 0.04). In the comparison between the DM + CAR and the DM group, *COL3A1* expression exhibited a substantial reduction of 1.94-fold (*p* < 0.01). All RT-qPCR analysis findings are presented in Figure 6.

## 3. Discussion

DN is an important complication that occurs in DM. It is the primary cause of end-stage renal disease and chronic renal failure; concurrently, it is reported that about one-third of diabetic individuals would ultimately obtain a diagnosis of DN [13]. Oxidative damage is associated with the pathogenesis of DN, and the effectiveness of various antioxidant therapies in its treatment has been reported in the literature [14]. CAR is one of the antioxidants commonly used in experimental models of DM [11,15]. This study demonstrated that, owing to its anti-inflammatory and antioxidant abilities, CAR significantly reduced the high blood urea and creatinine levels associated with DN. CAR also restored the levels of the antioxidant enzymes PON1 and ARES, which had diminished due to DM. Histopathological, immunohistochemical, and RT-qPCR assessments further confirmed its efficacy in mitigating diabetes-induced kidney injury.

Hyperglycemia induces oxidative stress and inflammatory responses that disrupt cellular membrane permeability and cause glomerular damage, ultimately leading to the accumulation of nitrogenous waste products. Increased ROS levels are significant contributors to this mechanism [1]. Blood levels of urea and creatinine, which are indicators of renal injury, have been reported to be increased in DN [16,17]. Several studies have demonstrated that antioxidant agents can be used to improve these parameters in DN [1,3]. For instance, it was reported that CAR normalized increased blood urea nitrogen and creatinine levels in diabetic rats [6]. The current study also revealed that urea and creatinine levels were significantly elevated in diabetic rats, while CAR administration improved both parameters. These findings align with the current literature. The beneficial effects of CAR are thought to be attributable to its ability to reduce the inflammatory response and increased ROS levels in DN, thereby maintaining cellular membrane integrity.

Human serum PON1 is an enzyme linked to high-density lipoprotein (HDL) and demonstrates significant antioxidant activity. PON1 and ARES are esterase enzymes expressed by the same gene and possess similar active sites. Ayan et al. reported that the activities of both PON1 and ARES were decreased in diabetic patients, with a more pronounced reduction observed in those with incipient DN compared to diabetic patients without nephropathy [18]. In the present study, PON1 and ARES levels were similarly reduced in diabetic rats; however, treatment with CAR reversed this diabetes-induced decline. CAR appears to enhance the activities of PON1 and ARES by diminishing oxidative stress, free radicals, and inflammation.

DM causes many histological abnormalities in the kidney, facilitating the development of DN. The principal histological alterations in DN encompass damage to both glomeruli and tubules. STZ-induced DM is linked to many renal modifications, including glomerular basement membrane thickening, glomerulosclerosis, expansion of the Bowman’s capsule space, tubular degeneration, and vacuolization [19]. Numerous antioxidants, including N-acetylcysteine and coenzyme Q10, have demonstrated efficacy in alleviating renal injury caused by oxidative stress [20,21,22]. CAR has also demonstrated efficacy in reversing histopathological alterations associated with DM [23]. The current study demonstrated that CAR treatment ameliorated the histological alterations generated by DM and considerably decreased the elevated histopathology scores seen in the DM group. By reducing oxidative stress and enhancing antioxidant defense mechanisms, CAR may preserve glomerular and tubular integrity, thereby attenuating diabetes-related renal damage.

The current study revealed that, with the noted histological changes, CAR administration reduced the increased fibrosis related to DM, as indicated by MT staining. Moreover, in accordance with the literature, the expression levels of *COL1A1* and *COL3A1*—both indicators of fibrosis—were increased in the DM group [24,25,26]. CAR therapy significantly reduced the DM-induced overexpression of these genes. CAR mitigates renal fibrosis by altering profibrotic signaling pathways and improving the oxidative and inflammatory microenvironment.

Oxidative stress and inflammation caused by hyperglycemia can initiate apoptosis, resulting in renal damage and, eventually, organ failure. Consequently, mitigating heightened oxidative stress and inflammation is a crucial approach for averting programmed cell death in DN [24]. The present study revealed that STZ-induced DM in rats resulted in increased renal apoptosis. Either ihc analysis or RTq-PCR analysis demonstrated a significant elevation of *Bax*, a pro-apoptotic marker, and a reduced expression of *Bcl-2* in the DM group. Furthermore, CAR treatment significantly reduced the expression of *Bax* while enhancing the levels of the anti-apoptotic protein Bcl-2 in renal tissue. Consistent with our findings, several antioxidants reported in the literature have demonstrated the ability to restore the disturbed apoptotic balance in STZ-induced DN through the modulation of *Bax* and *Bcl-2* expression [27,28]. This study revealed by ihc evaluation and RT-qPCR analysis that apoptosis is a crucial pathogenic pathway in DN and that CAR could exert its therapeutic effects by modifying apoptosis-related pathways, therefore aiding in the amelioration of DN.

In DN, the Nrf-2 pathway acts as an essential intrinsic cytoprotective mechanism that maintains redox homeostasis [29]. The suppression of Nrf-2 in experimental diabetes models exacerbates hyperglycemia-induced kidney damage by intensifying oxidative stress and inflammatory responses, whereas pharmacological activation of Nrf-2 is linked to decreased disease burden [30,31]. Nrf-2 signaling is mechanistically involved in critical downstream events, including apoptosis and fibrosis. Although our findings suggest that Nrf-2 signaling may contribute to the observed renoprotective effects, a more comprehensive understanding could be achieved by simultaneously evaluating the expression of key regulators, such as HO-1 and Keap-1. This limitation in our study highlights the need for further investigations to fully elucidate the mechanistic role of Nrf-2 in renal protection.

The biological effects of CAR identified in this study align with prior findings for other monoterpenoids often contained in essential oils. Phenolic monoterpenes, such as thymol, demonstrate similar antioxidant and anti-inflammatory effects, including decreases in lipid peroxidation indicators and enhancements in histopathological damage in experimental organ injury models [32,33]. Non-phenolic monoterpenes, such as *p*-cymene, have been documented to influence oxidative stress and inflammatory responses, but often with reduced efficacy [34]. Monoterpene alcohols, including linalool and geraniol, have exhibited protective properties against experimentally induced tissue damage, reinforcing the idea that monoterpenoids possess analogous cytoprotective mechanisms [35,36]. The protective benefits of CAR in our model seem to be indicative of a wider pharmacological profile common to essential oil monoterpenoids, rather than phenomena particular to the component alone.

## 4. Materials and Methods

This study was approved by the Animal Experiments Local Ethics Committee at Necmettin Erbakan University (decision date: 6 July 2022; decision number: 2022-030). The study was carried out in compliance with institutional protocols and international standards for the care and use of laboratory animals.

### 4.1. Animals and Experimental Groups

Wistar albino rats used in this study were 4 months old and weighed 250–300 g. The animals were provided with a combination of tap water and standard laboratory food, accessible ad libitum. The rats were housed in a room maintained at a temperature of 24 ± 1 °C and a humidity level of 45 ± 5%. Additionally, the room was kept on a 12 h light/dark cycle. A total of 36 rats were included in the study, of which nine were randomly allocated to the control group. Experimental type 1 DM was created in the remaining animals by an intraperitoneal (i.p) injection of STZ (50 mg/kg) (Sigma-Aldrich: S0130-1G, St. Louis, MO, USA), dissolved in 0.01 M sodium citrate buffer (pH 4.5) [37,38]. 72 h after STZ injection, a blood sample was taken from the tail vein for testing. A blood glucose level of 270 mg/dL or above was defined as diabetes. Following confirmation of hyperglycemia, animals meeting the predefined criteria were randomly assigned into experimental groups, excluding the control group. All four groups included an equal number of animals in the final analysis (*n* = 9 for each group):

Group 1: Control; no intervention was administered.

Group 2: DM; type 1 DM was generated.

Group 3: DM + DMSO; type 1 DM was generated as in group 2, and 0.1% dimethyl sulphoxide (DMSO) was administered.

Group 4: DM + CAR; type 1 DM was generated, and CAR was administered.

Blood glucose measurements were repeated weekly for confirmation and monitoring purposes. Additionally, 0.1% DMSO (0.1%, Sigma-Aldrich: Lot; SZBF3010V, St. Louis, MO, USA) was administered i.p daily for 4 weeks to animals in group 3. CAR at a dosage of 20 mg/kg (Purity: 98%, Sigma-Aldrich: Lot; SHBL6147, St. Louis, MO, USA), dissolved in 0.1% DMSO, was administered i.p daily for 4 weeks in group 4. The dosage of CAR was established based on the existing literature [39].

At the conclusion of the 4-week experiment, blood samples from the rats were obtained by cardiac puncture under anesthesia, which was induced by an injection of ketamine HCl and xylazine HCl at doses of 50 mg/kg and 10 mg/kg, respectively. Following this, all rats were sacrificed through cervical dislocation.

### 4.2. Biochemical Analysis

Upon conclusion of the experiment, blood samples obtained from all experimental animals in gel biochemistry tubes were subjected to centrifugation at 1500× *g* for 10 min to isolate the supernatant serum fraction. The obtained serum samples were subsequently aliquoted into Eppendorf tubes and stored at −80 °C until analysis. Serum samples were sent to the biochemistry laboratory, labeled without group identifiers and analyzed blindly by the investigator. All serum samples were concurrently thawed and equilibrated at room temperature before analysis. The materials were thereafter combined utilizing a vortex apparatus to prepare them for biochemical examination. Biochemical parameters such as urea and creatinine were measured colorimetrically on an autoanalyzer (Beckmann Coulter AU5800, Brea, CA, USA).

The activity of Paraoxonase (PON1) was quantified spectrophotometrically according to the methodology established by Eckerson et al. [40]. The test relies on the hydrolysis of paraoxon (diethyl *p*-nitrophenyl phosphate) into *p*-nitrophenol, measured by the increase in absorbance at 412 nm. Enzymatic activity was quantified in U/L utilizing the molar absorptivity coefficient of *p*-nitrophenol (ε = 18,000 M^−1^ cm^−1^). Measurements were conducted using a Beckman Coulter AU-680 autoanalyzer (Beckmann Coulter, Brea, CA, USA). The approach demonstrated high sensitivity, with a detection limit of 6.5 U/L and a linear range of 6.5 to 300 U/L.

Arylesterase (ARES) activity was quantified spectrophotometrically by monitoring the increase in absorbance at 270 nm, resulting from the enzymatic hydrolysis of phenylacetate (Sigma Aldrich, St. Louis, MO, USA) to phenol, as delineated by Eckerson et al. [40]. Following a 20 s delay, absorbance was measured for one minute. The enzyme activity was determined utilizing the molar absorptivity of phenol (ε = 1310 M^−1^ cm^−1^). Measurements were conducted using a Shimadzu UV-1700 spectrophotometer (Shimadzu, Kyoto, Japan). The approach exhibited a detection limit of 2.5 U/L, a quantification limit of 3.3 U/L, and a linear range from 2.5 to 91 U/L.

PON1 and ARES are enzymes that belong to the esterase group, encoded by the same gene and featuring similar active sites. Both PON1 and ARES can hydrolyze organophosphates, aryl halides, and alkyl halides. The PON1 enzyme acts as an antioxidant by preventing the oxidation of low-density lipoprotein (LDL) and neutralizing free radicals like hydrogen peroxide. ARES is recognized as an indicator of the primary protein, remaining stable despite variations in PON1 levels [41].

### 4.3. Histopathological Analysis

Subsequently to blood collection, the rats were sacrificed, and their abdominal cavities were incised for morphological evaluation of the kidneys. The renal tissues were then preserved in 10% neutral buffered formalin for histological examination. Tissue blocks fixed in paraffin were sectioned to a thickness of 5 µm utilizing a microtome. These sections were stained by using PAS, MT, and H&Estains.

H&E staining was performed to evaluate glomerular and tubular damage; sections were subjected to xylene treatment (three times for 20 min each) and a descending sequence of alcohol concentrations (100%, 96%, and 70%) for deparaffinization. The specimens were subsequently stained using Harris hematoxylin for 4 min and counterstained with 1% aqueous eosin for 2 min. PAS staining was performed to assess mesangial expansion and the thickening of the basement membrane; sections are stained with PAS for 5 min, and the periodic acid in the stain reacts with carbohydrates in the tissue, subsequently producing a purple-magenta hue using Schiff reagent for 5 min. Ultimately, hematoxylin is used to stain the nuclei. MT was used to determine collagen deposition for fibrosis assessment. Collagen is seen in green.

Histopathological scoring was conducted with the H&E and PAS techniques to assess glomerular and tubular damage, characterized by an enlarged urinary space, tubular dilatation, infiltration of inflammatory cells, glomerular basement membrane thickening, and an enlarged, disordered mesangial area. Histopathological alterations were classified as nonexistent (0), mild (1), moderate (2), and severe (3) [42]. The Zeiss Lab. A1 light microscope was utilized. Histopathological investigation was performed with a double-blind methodology. 

### 4.4. Immunohistochemistry (IHC) Analysis

Xylene was employed for deparaffinization for 30 min. After antigen retrieval, endogenous peroxidase activity was suppressed with 3% hydrogen peroxide. Super Block (ScyTek Laboratories, Logan, UT, USA) inhibited nonspecific antigen binding for 10 min. The sections were incubated overnight with Anti-B-cell lymphoma 2 (Bcl-2) (1:500; mouse monoclonal antibody, sc-7382, Santa Cruz Biotechnology, Dallas, TX, USA) and Anti-Bcl-2-associated X protein (Bax) (1:100; mouse monoclonal antibody, sc-23959, Santa Cruz Biotechnology) primary antibodies. The portions were treatment with a secondary antibody for 20 min. Streptavidin–peroxidase was employed to identify the antigen–antibody complex for 20 min. 3,3′-Diaminobenzidine (DAB) chromogen was administered for 15 min. Mayer’s hematoxylin was utilized for counterstaining for 5 min. Subsequently to staining, the sections were evaluated based on the following criteria: (0) no; (1) mild; (2) moderate; (3) severe staining [43].

### 4.5. Immunofluorescence (IF) Analysis

Sections of 5 μm in thickness on lysine slides were deparaffinized and dehydrated. Antigen retrieval with citrate buffer and %3 hydrogen peroxide applied. Super Block applied for 30 min. The slides were incubated at +4 °C overnight with Anti-NRF2 Rabbit pAb (1/1000; GB113808, Servicebio, Wuhan, China). The next day, a secondary antibody, Goat Anti-Rabbit IgG H&L (1/500; Alexa Fluor^®^ 488, ab150077, Abcam, Cambridge, UK) was used for 1 h at room temperature. After cleaning with PBS, slides were covered with a medium containing PBS, glycerine and Hoechst 33342 (ThermoFisher Scientific, Waltham, MA, USA) and observed with a Zeiss Axio microscope (Axiocam 212 color camera) (Zeiss, Oberkochen, Germany). The intensity mean value observed with Zeiss software ZEN 3.12 (Zen lite).

### 4.6. RNA Extraction, cDNA Synthesis, and Gene Expression Analysis by RT-qPCR

Total RNA isolation was first performed from kidney tissues for gene expression profiling using GeneAll RiboEX (301-001, GeneAll, Seoul, Republic of Korea). DNase I enzyme (Thermo Fisher Scientific, Waltham, MA, USA, #EN0521) was utilized to eliminate DNA from the extracted RNA samples. The RNA samples’ purity and quality were assessed at 260 and 280 nm with a Nanodrop spectrophotometer. Subsequently, 1 µg of total RNA was transcribed into cDNA utilizing a cDNA synthesis kit (BIO-RAD, Hercules, CA, USA, 170-8891). The primers for the target genes examined in the study were developed via the IDT PrimerQuest online software (idtdna, https://www.idtdna.com/PrimerQuest/Home/Index, accessed on 29 October 2025) (Table 1). GAPDH served as the housekeeping gene for normalizing gene expression levels. The mRNA expression levels of the target genes *BAX*, BCL2, *COL1A1*, and *COL3A1* were quantified using the 5x HOT FIREPol^®^ EvaGreen^®^ qPCR Master Mix Plus (ROX) from Solis BioDyne (Tartu, Estonia). The BIO-RAD CFX Connect device employed PCR settings that included an initial activation phase at 95 °C for 12 min, followed by 42 amplification cycles at 95 °C for 15 s, 60 °C for 25 s, and 72 °C for 25 s. Tissue samples were labeled in a manner that prevented the researcher performing RT-qPCR analysis from knowing the group identity, ensuring a blind assessment.

### 4.7. Statistical Analysis

Statistical analysis was conducted using GraphPad Prism (version 8.4.2). The normality of the data from the groups was evaluated with the Shapiro–Wilk test. Continuous variables were expressed as the mean ± SD. The one-way ANOVA test was performed to assess normally distributed numerical data among groups (*n* = 9 samples per group). The variance homogeneity was evaluated, and a post hoc analysis was conducted. The post hoc Tukey’s test was utilized for pairwise comparisons following ANOVA. The data that did not exhibit a normal distribution were analyzed using the Kruskal–Wallis test with *n* = 9 samples per group, while differences between matched groups were assessed with Dunn’s test. The assessment of relative gene expression was conducted utilizing the 2^−∆∆CT^ technique (*n* = 9 samples per group). A *p*-value below 0.05 was accepted for statistical significance. Sample size was determined a prior by power analysis (one-way ANOVA, α = 0.05, power = 0.80) using pilot estimates of variance; resulting sample size was *n* = 9 per group. Power analysis was performed using the G*Power software (version 3.1.9.4).

## 5. Conclusions

This study revealed that CAR effectively mitigates STZ-induced DM. CAR seems to be a potential therapeutic agent for DN, demonstrating enhancements in several parameters, including biochemical, histological, immunohistochemical, and molecular (RT-qPCR) data. Nevertheless, further investigations are warranted to clarify the efficacy of different doses and long-term effects of CAR and to better define the underlying biological processes involved in its protective action. In addition, future experimental and clinical studies may help determine its potential therapeutic value and applicability in the prevention and treatment of diabetic kidney disease.

## Figures and Tables

**Table 1:** Primary sequences of target and reference genes used in RT-qPCR analysis.

Gene	Forward Primary (5′-3′)	Reverse Primary (5′-3′)
*BAX*	GATGGCCTCCTTTCCTACTTC	CTTCTTCCAGATGGTGAGTGAG
*BCL-2*	GGAGGATTGTGGCCTTCTTT	GTCATCCACAGAGCGATGTT
*COL1A1*	CCAATGGTGCTCCTGGTATT	GTTCACCACTGTTGCCTTTG
*COL3A1*	GTGTGATGATGAGCCACTAGAC	TGACAGGAGCAGGTGTAGAA
*GAPDH*	GCATTGCAGAGGATGGTAGAG	GCGGGAGAAGAAAGTCATGATTAG

## Data Availability

The original contributions presented in this study are included in the article. Further inquiries can be directed to the corresponding author.

## References

[1] Jin Q., Liu T., Qiao Y., Liu D., Yang L., Mao H., Ma F., Wang Y., Peng L., Zhan Y. (2023). Oxidative Stress and Inflammation in Diabetic Nephropathy: Role of Polyphenols. Front. Immunol..

[2] Ekinci B., Suleyman B., Mammadov R., Gezer A., Mendil A.S., Akbas N., Bulut S., Dal C.N., Suleyman H. (2022). The Effect of Carvacrol upon Experimentally Induced Diabetic Neuropathy and Neuropathic Pain in Rats. Acta Pol. Pharm. Drug Res..

[3] Darenskaya M., Kolesnikov S., Semenova N., Kolesnikova L. (2023). Diabetic Nephropathy: Significance of Determining Oxidative Stress and Opportunities for Antioxidant Therapies. Int. J. Mol. Sci..

[4] Dwivedi S., Sikarwar M.S. (2025). Diabetic Nephropathy: Pathogenesis, Mechanisms, and Therapeutic Strategies. Horm Metab Res..

[5] Li X., Lu L., Hou W., Huang T., Chen X., Qi J., Zhao Y., Zhu M. (2022). Epigenetics in the pathogenesis of diabetic nephropathy. Acta Biochim. Biophys. Sin..

[6] Jamshidi H.R., Zeinabady Z., Zamani E., Shokrzadeh M., Shaki F. (2018). Attenuation of Diabetic Nephropathy by Carvacrol through Anti-Oxidative Effects in Alloxan-Induced Diabetic Rats. Res. J. Pharmacogn..

[7] Imran M., Aslam M., Alsagaby S.A., Saeed F., Ahmad I., Afzaal M., Arshad M.U., Abdelgawad M.A., El-Ghorab A.H., Khames A. (2022). Therapeutic Application of Carvacrol: A Comprehensive Review. Food Sci. Nutr..

[8] Vassiliou E., Awoleye O., Davis A., Mishra S. (2023). Anti-Inflammatory and Antimicrobial Properties of Thyme Oil and Its Main Constituents. Int. J. Mol. Sci..

[9] Kowalczyk A., Przychodna M., Sopata S., Bodalska A., Fecka I. (2020). Thymol and Thyme Essential Oil-New Insights into Selected Therapeutic Applications. Molecules.

[10] Costa M.F., Durço A.O., Rabelo T.K., Barreto R.S.S., Guimarães A.G. (2019). Effects of Carvacrol, Thymol and essential oils containing such monoterpenes on wound healing: A systematic review. J. Pharm. Pharmacol..

[11] Li Y., Mai Y., Qiu X., Chen X., Li C., Yuan W., Hou N. (2020). Effect of Long-Term Treatment of Carvacrol on Glucose Metabolism in Streptozotocin induced Diabetic Mice. BMC Complement. Med. Ther..

[12] Hoca M., Becer E., Vatansever H.S. (2024). Carvacrol is potential molecule for diabetes treatment. Arch. Physiol. Biochem..

[13] Tanase D.M., Gosav E.M., Anton M.I., Floria M., Seritean Isac P.N., Hurjui L.L., Tarniceriu C.C., Costea C.F., Ciocoiu M., Rezus C. (2022). Oxidative Stress and NRF2/KEAP1/ARE Pathway in Diabetic Kidney Disease (DKD): New Perspectives. Biomolecules.

[14] Hernandez L.F., Eguchi N., Whaley D., Alexander M., Tantisattamo E., Ichii H. (2022). Anti-Oxidative Therapy in Diabetic Nephropathy. Front. Biosci. Sch..

[15] Bayramoglu G., Senturk H., Bayramoglu A., Uyanoglu M., Colak S., Ozmen A., Kolankaya D. (2014). Carvacrol Partially Reverses Symptoms of Diabetes in STZ-Induced Diabetic Rats. Cytotechnology.

[16] Mohany M., Ahmed M.M., Al-Rejaie S.S. (2022). The Role of NF-κB and Bax/Bcl-2/Caspase-3 Signaling Pathways in the Protective Effects of Sacubitril/Valsartan (Entresto) against HFD/STZ-Induced Diabetic Kidney Disease. Biomedicines.

[17] Wahab N.A.A., Giribabu N., Kilari E.K., Salleh N. (2022). Abietic Acid Ameliorates Nephropathy Progression via Mitigating Renal Oxidative Stress, Inflammation, Fibrosis and Apoptosis in High Fat Diet and Low Dose Streptozotocin-Induced Diabetic Rats. Phytomedicine.

[18] Ayan D., Şeneş M., Çaycı A.B., Söylemez S., Eren N., Altuntaş Y., Öztürk F.Y. (2019). Evaluation of Paraoxonase, Arylesterase, and Homocysteine Thiolactonase Activities in Patients with Diabetes and Incipient Diabetes Nephropathy. J. Med. Biochem..

[19] Chen H.W., Yang M.Y., Hung T.W., Chang Y.C., Wang C.J. (2019). Nelumbo Nucifera Leaves Extract Attenuate the Pathological Progression of Diabetic Nephropathy in High-Fat Diet-Fed and Streptozotocin-Induced Diabetic Rats. J. Food Drug Anal..

[20] Chen Y.J., Kong L., Tang Z.Z., Zhang Y.M., Liu Y., Wang T.Y., Liu Y.W. (2019). Hesperetin Ameliorates Diabetic Nephropathy in Rats by Activating Nrf2/ARE/Glyoxalase 1 Pathway. Biomed. Pharmacother..

[21] Mahajan M.S., Upasani C.D., Upaganlawar A.B., Gulecha V.S. (2020). Renoprotective Effect of Co-Enzyme Q10 and N-Acetylcysteine on Streptozotocin-Induced Diabetic Nephropathy in Rats. Int. J. Diabetes Clin. Res..

[22] Canbaz F.A., Yurtçu M., Oltulu P., Taştekin G., Kocabaş R., Doğan M. (2024). Investigation of the Effects of N-Acetylcysteine and Selenium on Vesicoureteral Reflux Nephropathy: An Experimental Study. J. Pediatr. Surg..

[23] Sun Y., Qu H., Niu X., Li T., Wang L., Peng H. (2024). Carvacrol Improves Blood Lipid and Glucose in Rats with Type 2 Diabetes Mellitus by Regulating Short-Chain Fatty Acids and the GPR41/43 Pathway. Korean J. Physiol. Pharmacol..

[24] Zhang Z., Huang Q., Zhao D., Lian F., Li X., Qi W. (2023). The Impact of Oxidative Stress-Induced Mitochondrial Dysfunction on Diabetic Microvascular Complications. Front. Endocrinol..

[25] Patel S.H., Sabbaghi A., Carroll C.C. (2018). Streptozotocin-Induced Diabetes Alters Transcription of Multiple Genes Necessary for Extracellular Matrix Remodeling in Rat Patellar Tendon. Connect. Tissue Res..

[26] Diao X., Shen E., Wang X., Hu B. (2011). Differentially Expressed MicroRNAs and Their Target Genes in the Hearts of Streptozotocin-Induced Diabetic Mice. Mol. Med. Rep..

[27] El Azab E.F., Mostafa H.S. (2022). Geraniol Ameliorates the Progression of High Fat-Diet/Streptozotocin-Induced Type 2 Diabetes Mellitus in Rats via Regulation of Caspase-3, Bcl-2, and Bax Expression. J. Food Biochem..

[28] Guo J., Li J., Wei H., Liang Z. (2021). Maackiain Protects the Kidneys of Type 2 Diabetic Rats via Modulating the Nrf2/Ho-1 and Tlr4/Nfκb/Caspase-3 Pathways. Drug Des. Dev. Ther..

[29] Gupta A., Behl T., Sehgal A., Bhatia S., Jaglan D., Bungau S. (2021). Therapeutic potential of Nrf-2 pathway in the treatment of diabetic neuropathy and nephropathy. Mol. Biol. Rep..

[30] Alshehri A.S. (2023). Kaempferol attenuates diabetic nephropathy in streptozotocin-induced diabetic rats by a hypoglycaemic effect and concomitant activation of the Nrf-2/Ho-1/antioxidants axis. Arch. Physiol. Biochem..

[31] Zaghloul R.A., Abdelghany A.M., Samra Y.A. (2022). Rutin and selenium nanoparticles protected against STZ-induced diabetic nephropathy in rats through downregulating Jak-2/Stat3 pathway and upregulating Nrf-2/HO-1 pathway. Eur. J. Pharmacol..

[32] Wang Q., Qi G., Zhou H., Cheng F., Yang X., Liu X., Wang R. (2023). Protective effect of thymol on glycerol-induced acute kidney injury. Ren. Fail..

[33] Saravanan S., Pari L. (2016). Protective effect of thymol on high fat diet induced diabetic nephropathy in C57BL/6J mice. Chem. Biol. Interact..

[34] Peirovy Y., Asle-Rousta M. (2024). Thymol and p-Cymene Protect the Liver by Mitigating Oxidative Stress, Suppressing TNF-α/NF-κB, and Enhancing Nrf2/HO-1 Expression in Immobilized Rats. Chem. Biol. Drug Des..

[35] Deepa B., Venkatraman Anuradha C. (2013). Effects of linalool on inflammation, matrix accumulation and podocyte loss in kidney of streptozotocin-induced diabetic rats. Toxicol. Mech. Methods.

[36] El-Said Y.A.M., Sallam N.A.A., Ain-Shoka A.A., Abdel-Latif H.A. (2020). Geraniol ameliorates diabetic nephropathy via interference with miRNA-21/PTEN/Akt/mTORC1 pathway in rats. Naunyn Schmiedebergs Arch. Pharmacol..

[37] Cüce G., Sözen M.E., Çetinkaya S., Canbaz H.T., Seflek H., Kalkan S. (2015). Effects of Nigella Sativa L. Seed Oil on Intima–Media Thickness and Bax and Caspase 3 Expression in Diabetic Rat Aorta. Anatol. J. Cardiol..

[38] Kamli-Salino S.E.J., Brown P.A.J., Haschler T.N., Liang L., Feliers D., Wilson H.M., Delibegovic M. (2023). Induction of experimental diabetes and diabetic nephropathy using anomer-equilibrated streptozotocin in male C57Bl/6J mice. Biochem. Biophys. Res. Commun..

[39] Gültekin B., Çetinkaya Karabekir S., Ayan I.Ç., Savaş H.B., Cüce G., Kalkan S.S. (2025). Effect of Carvacrol on Diabetes-Induced Oxidative Stress, Fibrosis and Apoptosis in Testicular Tissues of Adult Rats. Physiol. Res..

[40] Eckerson H.W., Wyte C.M., La Du B.N. (1983). The Human Serum Paraoxonase/Arylesterase Polymorphism. Am. J. Hum. Genet..

[41] Akın F., Yazar A., Türe E., Gültekin Ü., Kılıç A.O., Topçu C., Odabaş D., Yorulmaz A. (2023). Paraoxonase-1 and Arylesterase Activities in Children with Acute Bronchiolitis. J. Contemp. Med..

[42] Cuce G., Cetinkaya S., Isitez N., Kuccukturk S., Sozen M.E., Kalkan S., Cigerci I.H., Demirel H.H. (2016). Effects of Curcumin on Methyl Methanesulfonate Damage to Mouse Kidney. Biotech. Histochem..

[43] Karabekir S.C., Gultekin B., Ayan I.C., Savas H.B., Cuce G., Kalkan S. (2024). Protective Effect of Astaxanthin on Histopathologic Changes Induced by Bisphenol A in the Liver of Rats. Pak. Vet. J..

